# Stem Cells and Cell-Free Therapies for Olfactory Epithelium Regeneration: Insights from Experimental Models

**DOI:** 10.3390/ijms26189024

**Published:** 2025-09-16

**Authors:** Keun-Ik Yi, Ji-Hwan Park, Sung-Dong Kim, Sue Jean Mun, Kyu-Sup Cho

**Affiliations:** 1 Department of Otorhinolaryngology, Pusan National University School of Medicine, Yangsan 50612, Republic of Korea; cross_lette@hanmail.net; 2 Department of Otorhinolaryngology and Research Institute for Convergence of Biomedical Science and Technology, Pusan National University Yangsan Hospital, Yangsan 50612, Republic of Korea; nobleivy@naver.com (J.-H.P.); baskie23@naver.com (S.J.M.); 3 Department of Otorhinolaryngology and Biomedical Research Institute, Pusan National University School of Medicine, Pusan National University Hospital, Busan 49241, Republic of Korea; applekims@daum.net

**Keywords:** stem cells, secretome, extracellular vesicles, anosmia, olfactory mucosa, nerve regeneration

## Abstract

Olfactory impairment is one of the most common diseases of the sense organs, and it is closely related to quality life. Although the molecular mechanism of olfaction was recently brought to light, the pathophysiology and effective treatments for olfactory dysfunction still remain challenging. Olfactory impairment can be caused by the degeneration of olfactory receptor neurons in the nose and also by the degeneration of the olfactory bulb of the olfactory cortex. Several studies have shown that stem cells promote the regeneration of the olfactory neuroepithelium after permanent damage in anosmic mice. Transplanted adipose stem cells differentiated into olfactory receptor neurons and endothelial cells. Recently, cell-free approaches using stem cell-derived secretome and extracellular vesicles (EVs) have emerged as a safer, more controllable alternative. These vesicles contain biologically active cargo such as neurotrophins, cytokines, and microRNAs that promote neurogenesis and modulate inflammation. Although direct application in anosmia models remains limited, findings from related neural injury models suggest that secretome- and EV-based therapies may achieve comparable regenerative efficacy to stem cell transplantation. This review summarizes current evidence on the regenerative capacity of stem cells and their secretome or EVs as therapeutic strategies for olfactory epithelium regeneration.

## 1. Introduction

Olfaction, or the sense of smell, is the sensory system that enables humans to detect and differentiate odors [1]. It plays a critical role in human experience, influencing not only the perception of flavors and environmental hazards but also contributing to emotional and social well-being [2]. Olfactory dysfunction—including hyposmia (reduced sense of smell) and anosmia (complete loss of smell)—is one of the most common and often overlooked disorders of the sensory system [3]. Individuals with olfactory deficits frequently experience reduced appetite, altered taste perception, decreased awareness of personal hygiene, and symptoms of depression and social isolation, all of which can significantly impair quality of life [3,4].

Olfactory dysfunction may arise from various causes, including viral infections of upper respiratory tract, chronic rhinosinusitis, traumatic brain injury, neurodegenerative diseases such as Parkinson’s and Alzheimer’s disease, and aging [4,5]. Conventional therapeutic approaches such as olfactory training, intranasal or systemic corticosteroids, and surgical interventions have been employed to address olfactory dysfunction. Although these treatments may offer temporary or partial relief, their effectiveness remains inconsistent and often limited, particularly in patients with extensive damage to the olfactory epithelium or significant neuronal degeneration [5,6].

Given the regenerative potential of the olfactory epithelium, research has increasingly focused on the role of resident stem cells in restoring function after injury. The olfactory system is unique among sensory systems in that it retains lifelong neurogenic capacity, primarily through two populations of progenitor cells: horizontal basal cells (HBCs) and globose basal cells (GBCs) [7]. Understanding how these stem cells contribute to the regeneration of the olfactory epithelium may facilitate the development of novel therapeutic strategies for patients suffering from persistent olfactory loss.

This review discusses recent advances in the stem cell biology within the olfactory epithelium, evaluates the therapeutic potential of exogenously administrated stem cell, and highlights emerging experimental models and technologies that may facilitate future clinical applications.

## 2. Olfactory Nervous System

The olfactory nervous system is a highly specialized sensory pathway responsible for the detection, transduction, and central processing of odorant stimuli. It comprises both peripheral and central structures, which together enable dynamic odor recognition and adaptation while maintaining the capacity for neurogenesis throughout life.

### 2.1. Peripheral Components: The Olfactory Epithelium

The olfactory epithelium, located in the dorsal-posterior region of the nasal cavity, is a specialized pseudostratified neuroepithelium composed of sustentacular cells, immature and mature olfactory sensory neurons (OSNs), and two distinct types of basal stem cells: HBCs and GBCs [7,8]. OSNs are bipolar neurons that extend apical dendrites bearing odorant receptor-expressing cilia into the nasal lumen, while their unmyelinated axons traverse the cribriform plate to synapse within the olfactory bulb [9]. The epithelial microenvironment is maintained by sustentacular cells, which provide metabolic and structural support, as well as by Bowman’s gland cells, which secrete mucus essential for odorant dissolution and epithelial protection [9]. HBCs and GBCs, located adjacent to the basal lamina, serve as progenitor cells that continuously regenerate OSNs throughout life. Although this peripheral neural structure possesses robust regenerative capacity, it remains vulnerable to irreversible damage under pathological conditions [10].

HBCs remain largely quiescent under homeostatic conditions and become activated primarily in response to severe epithelial injury. Upon activation, HBSs exhibit multipotent capacity, regenerating not only neurons but also non-neuronal cell types such as sustentacular and glandular cells, thereby playing a key role in epithelial repair. This process is regulated by transcription factors such as p63, which maintains HBC dormancy until activation signals are received [10,11]. In contrast, GBCs are actively proliferating progenitor cells responsible for the routine replacement of OSNs under normal physiologic conditions [10]. Their differentiation is governed by multiple signaling pathways, including the Wnt and Notch pathways, as well as transcription factors such as Sox2, Pax6, and Ascl1 [12,13]. The coordinated interplay between HBCs and GBCs is critical for balancing steady-state maintenance with injury-induced regeneration. However, their regenerative potential can be compromised by aging or chronic inflammation, which alters their proliferation and differentiation profiles [10]. Understanding the molecular mechanisms that regulate the activation, proliferation, and differentiation of these basal cells may provide a foundation for regenerative strategies aimed at restoring olfactory function in patients with anosmia.

### 2.2. Central Components: The Olfactory Bulb and Higher Brain Regions

Axons of mature OSNs converge onto discrete neuropil structures called glomeruli in the olfactory bulb, where they form synapses with second-order neurons, including mitral and tufted cells [14]. These projection neurons transmit olfactory information to various regions of the olfactory cortex through the lateral olfactory tract. Primary targets include the piriform cortex, which mediates odor perception and discrimination; the amygdala, which processes odor-related emotional responses; and the entorhinal cortex and hippocampus, which integrate olfactory cues with memory formation [15]. Additionally, the orbitofrontal cortex participates in conscious olfactory perception and decision-making. Uniquely, the olfactory system bypasses the thalamus in its primary pathway, allowing for direct influence on limbic and associative cortical areas [16].

### 2.3. Distinctive Characteristics of the Olfactory Nervous System

The olfactory system is characterized by several distinctive features that distinguish it from other sensory systems [1]. First, it maintains continuous neurogenesis throughout life—a capacity not observed in most other parts of the adult nervous system. This regenerative ability is supported by resident stem cells in the olfactory epithelium, particularly GBCs and HBCs, which are differentially activated depending on the extent of epithelial damage. Second, the olfactory epithelium is directly exposed to environmental agents such as toxins, pathogens, and particulate matter, rendering it particularly vulnerable to injury and degeneration. Third, the system exhibits structural and functional plasticity in response to sensory experience and environmental changes, as demonstrated by the remodeling of synaptic connections within the olfactory bulb. A comprehensive understanding of the olfactory nervous system’s structure and regenerative potential is foundational for developing stem cell-based therapies aimed at restoring function in individuals with olfactory dysfunction.

## 3. Experimental Models of Anosmia

Animal models of anosmia have been widely utilized to investigate the pathophysiology of olfactory dysfunction, the stem cell-mediated regeneration, and potential therapeutic interventions. These models typically aim to mimic olfactory dysfunction observed in clinical conditions such as post-viral anosmia, traumatic injury, or neurodegenerative disease. Several well-established methods are used to induce anosmia in rodents, each with specific advantages and limitations.

### 3.1. Chemical Ablation

Chemical ablation is one of the most widely used methods for inducing anosmia in animal models due to its simplicity, reproducibility, and selective injury to the olfactory epithelium. Various chemical agents have been employed to selectively damage OSNs and supporting epithelial cells, while often sparing the olfactory bulb (Table 1).

Methimazole, a thiourea derivative used clinically as an antithyroid drug, induces widespread apoptosis in the olfactory epithelium when administered systemically via intraperitoneal injection [17]. Its mechanism involves oxidative stress and mitochondrial dysfunction in both OSNs and basal progenitor cells [18]. Importantly, methimazole-induced damage is typically dose-dependent and reversible, and it spares the olfactory bulb, making it an ideal model for studying stem cell-mediated epithelial regeneration. Histologically, methimazole causes near-complete degeneration of mature OSNs and temporary depletion of globose basal cells, followed by robust regenerative activity from HBCs [17].

Zinc sulfate is commonly administered via intranasal instillation, where it acts locally to induce necrosis of epithelial cells through direct oxidative damage and membrane disruption [19]. Compared to methimazole, zinc sulfate produces a more variable and patchy injury, and its effects are highly dependent on dosage, concentration, and delivery technique. Although it is effective in causing acute anosmia, inconsistencies in epithelial damage and regeneration rates have limited its use in mechanistic regeneration studies. Zinc sulfate may also affect non-olfactory regions of the nasal cavity, including respiratory epithelium, making specificity a concern.

Dichlobenil, a herbicide that inhibits cellulose biosynthesis, has been used experimentally to induce targeted degeneration of sustentacular and supporting cells in the olfactory epithelium [20]. Its lipophilic nature enables it to be delivered via inhalation or intranasal routes, where it disrupts the structural integrity of the epithelial barrier. Although less commonly used than methimazole or zinc sulfate, dichlobenil offers a model of non-neuronal cell-targeted damage, allowing for exploration of sustentacular cell roles in neuroepithelial maintenance and regeneration.

3-methylindole (3-MI), a microbial metabolite of tryptophan, has been utilized in recent anosmia models to induce olfactory epithelial damage through systemic oxidative injury [21]. When administered intraperitoneally, 3-MI is metabolized into reactive intermediates that cause apoptosis of olfactory sensory neurons, epithelial disorganization, and inflammatory cell infiltration. Although traditionally used in pulmonary toxicity studies, its effects on the olfactory system are gaining attention as a model for inflammation-mediated olfactory dysfunction. Compared to methimazole, 3-MI induces broader systemic effects but offers value in studying both neuroepithelial degeneration and regeneration under inflammatory conditions.

### 3.2. Surgical and Mechanical Injury

Surgical and mechanical damage to the olfactory epithelium or olfactory bulb has been used to simulate trauma-induced anosmia in animal studies. Surgical removal of the olfactory bulbs (olfactory bulbectomy) leads to retrograde degeneration of OSNs and permanent anosmia [22]. Although this method eliminates the potential for olfactory recovery, it is highly effective for studying central nervous system responses, including neuroplasticity, behavioral adaptations, and compensatory circuit remodeling in the absence of olfactory input.

Another approach involves unilateral transection of the olfactory nerve, in which the olfactory nerve is surgically severed on one side, thereby disrupting axonal connections between the olfactory epithelium and the olfactory bulb [23]. This procedure induces selective degeneration of OSNs and epithelial remodeling on the lesioned side, while leaving the contralateral side intact as an internal control. Unlike olfactory bulbectomy, this method preserves the olfactory bulb, allowing for precise investigation of peripheral neurodegeneration, local immune responses, and stem cell-mediated epithelial repair without the confounding effects of central olfactory circuit disruption.

In contrast, mechanical disruption of the olfactory epithelium using fine brushes, scraping instruments, or nasal saline irrigation can produce localized, moderate injury while preserving the basal stem cell population [24]. These models are particularly well-suited for investigating the regenerative capacity of the olfactory epithelium under defined injury conditions, as they allow for subsequent spontaneous recovery and the histological analysis of epithelial regeneration.

### 3.3. Genetic Models

Transgenic mouse models have enabled precise, cell type-specific manipulation of OSNs and basal stem cells, providing powerful tools for studying the molecular mechanisms underlying olfactory neurogenesis and epithelial maintenance [25]. Two primary genetic strategies are commonly employed: conditional cell ablation and gene knockout models.

Conditional ablation typically utilizes the Cre-loxP recombination system under the control of cell-specific promoters such as *OMP-Cre* (for mature OSNs) or *K5-CreER* (for basal cells) [26]. This approach allows for temporally and spatially controlled deletion of target genes or cell populations, enabling researchers to assess the contribution of specific cell types to epithelial homeostasis and regeneration.

In parallel, knockout mouse models lacking key transcription factors essential for olfactory epithelial development and regeneration—such as *Sox2*, *Ascl1*, and *p63*—have been used to generate congenital or progressive anosmia phenotypes [27]. These models are especially valuable for dissecting gene-specific roles in stem cell maintenance, neuronal lineage commitment, and response to injury. Together, these genetic tools facilitate mechanistic studies of olfactory neurobiology and offer experimental systems to test therapeutic gene targets for olfactory dysfunction.

### 3.4. Infection and Inflammation-Induced Models

Viral infections—such as influenza and SARS-CoV-2—as well as chronic inflammation have been strongly implicated in the development of anosmia in both clinical and experimental settings [28]. Recent animal models replicate these conditions using intranasal inoculation of respiratory viruses, including Sendai virus and mouse-adapted coronaviruses, to induce inflammation-mediated damage to OSNs and supporting cells within the olfactory epithelium [29].

In parallel, lipopolysaccharide (LPS)-induced inflammation is employed to model chronic rhinosinusitis [30]. Intranasal LPS application triggers robust local immune responses, disrupting epithelial barrier function, altering the stem cell niche, and impairing epithelial regeneration. These models are especially valuable for investigating the interplay between the immune system and olfactory stem cell activity, as well as the mechanisms underlying prolonged or irreversible olfactory dysfunction.

## 4. Effect of Stem Cells on Olfactory Epithelium Regeneration

The regenerative process of the olfactory epithelium is a tightly regulated, multi-step phenomenon that involves stem cell activation, proliferation, differentiation, and integration of newly formed OSNs into functional neural circuits [31]. Recent research has deepened our understanding of the intrinsic and extrinsic factors governing this regenerative process, highlighting the therapeutic potential of both endogenous and exogenous stem cells for treating olfactory dysfunction.

### 4.1. Endogenous Stem Cell-Mediated Regeneration

Under physiological conditions, GBCs function as the primary progenitor population responsible for the continuous generation of new OSNs [10,32]. Following injury, HBCs become activated and contribute to the regeneration of not only neuronal but also supporting epithelial cells [10,33]. Studies using lineage-tracing and cell fate-mapping have demonstrated that GBCs, characterized by the expression of transcription factors such as *Ascl1*, *Sox2*, and *Pax6*, undergo transient amplification followed by neuronal differentiation to replenish the population of lost OSNs [34]. However, HBCs, which typically remain in a quiescent state under homeostatic conditions and express markers including *p63* and *Krt5*, are triggered by severe epithelial injury to acquire a multipotent phenotype [35]. These cells subsequently generate diverse progeny, including GBCs, sustentacular cells, and mature OSNs, thereby contributing to epithelial repair.

The activation and differentiation of these endogenous stem cell populations are tightly regulated by several key signaling pathways. Notch signaling is essential for maintaining HBC quiescence and preventing premature neuronal differentiation [36]. Wnt/β-catenin signaling plays a critical role in promoting progenitor cell proliferation and commitment toward the OSN lineage [37], while bone morphogenic protein (BMP) and fibroblast growth factor (FGF) pathways orchestrate spatial patterning and determine cellular identity during the regeneration process [38]. Disruption of these regulatory pathways due to chronic inflammation, aging, or genetic defects impairs the regenerative capacity of the olfactory epithelium.

### 4.2. Molecular Signaling in Stem Cell-Mediated Regeneration

**Figure 1:**
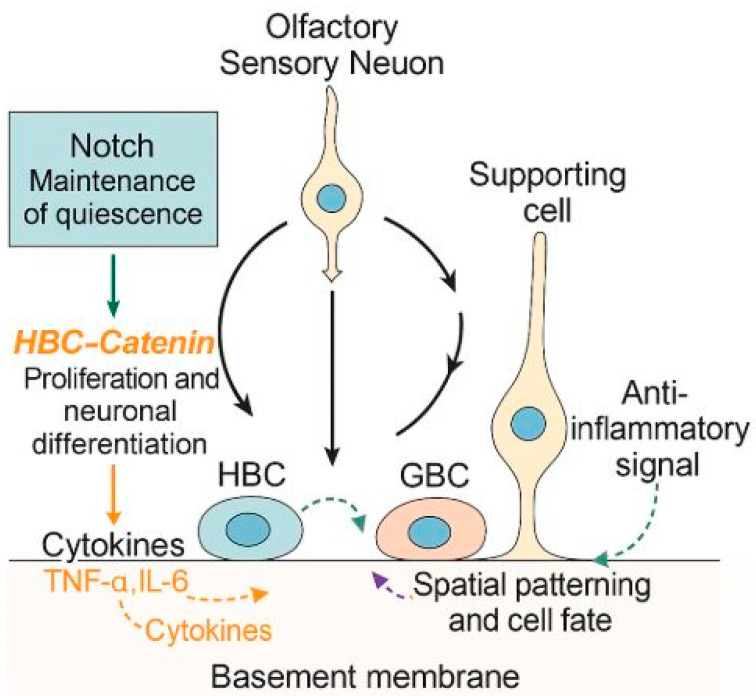
Graphical illustration of stem cell-mediated regeneration and molecular signaling in the olfactory epithelium. Under normal conditions, globose basal cells (GBCs) continuously replenish olfactory sensory neurons, while horizontal basal cells (HBCs) remain quiescent. Following injury, HBCs are activated and differentiate into neuronal and non-neuronal cell types. Key signaling pathways, including Notch (maintenance of quiescence), Wnt/β-catenin (proliferation and neuronal differentiation), restriction of neurogenesis, and spatial patterning and cell fate, regulate this regenerative process. Inflammatory cytokines (e.g., TNF-α, IL-6) impair regeneration, whereas anti-inflammatory signals (e.g., TGF-β) restore a permissive environment.

Stem cell activity in the olfactory epithelium is tightly orchestrated by a complex network of intrinsic transcriptional programs and extrinsic signals derived from the local niche. Growth factors such as FGF2, epidermal growth factor (EGF), and insulin-like growth factor (IGF)-1 stimulate basal cell proliferation and promote neuronal lineage progression following injury [39]. Morphogen pathways, including Wnt/β-catenin signaling, support progenitor self-renewal [37], whereas BMP and Notch pathways act to restrict excessive neurogenesis and ensure the balanced production of neuronal and non-neuronal cell types [36,38]. Immune-derived cytokines, particularly pro-inflammatory mediators such as TNF-α and IL-6, can impair basal cell proliferation and differentiation, while anti-inflammatory cytokines and TGF-β signaling contribute to the restoration of a permissive regenerative environment [40]. Interactions between stem cells and extracellular matrix components, including laminin and integrins, further influence cell adhesion, orientation, and fate specification during tissue repair [41]. The disruption of these regulatory mechanisms, as occurs with viral infections, chronic inflammation, or age-related degeneration, compromises the capacity of stem cells to regenerate a functional olfactory epithelium, leading to persistent sensory deficits. A graphical summary of these processes, including the activation of HBCs and GBCs and the major signaling pathways involved in olfactory epithelium regeneration, is provided in Figure 1.

### 4.3. Exogenous Stem Cell Therapy

**Figure 2:**
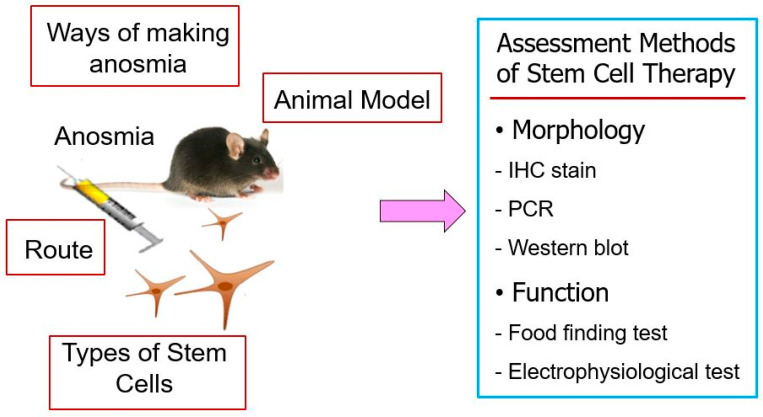
Schematic overview of stem cell therapy in animal models of anosmia. Previous studies for stem cell therapy in anosmia animal model differ in the methods for inducing anosmia in animal models, types of stem cell, administration routes of stem cells, and assessment methods for evaluating treatment effectiveness.

Stem cells are undifferentiated cells that have the potential to develop into many different cell types. They have been reported to possess the ability to self-renew in an undifferentiated state and to differentiate into many types of cells with specific functions upon receiving appropriate triggers [42]. Both embryonic and adult stem cells have been studied as a promising source for therapeutic applications in the repair of damaged tissues and regenerative medicine [43,44]. Exogenous stem cell therapy has emerged as a promising experimental approach to enhance olfactory epithelium regeneration when endogenous repair mechanisms are insufficient. Several studies have investigated the transplantation of different stem cell sources into animal models of anosmia, reporting varying degrees of structural and functional recovery (Table 2) [45,46,47,48,49]. These studies differ in their choice of methods for inducing anosmia in animal models, stem cell populations, administration routes of stem cells, and assessment methods for evaluating treatment effectiveness, reflecting the complexity of developing standardized therapeutic protocols (Figure 2).

#### 4.3.1. Animal Model of Olfactory Dysfunction

Various animal models have been employed to mimic olfactory dysfunction prior to stem cell transplantation. Kim et al. established a transection model in rats by severing the olfactory nerve unilaterally, thereby inducing degeneration of the olfactory epithelium [45]. Other studies have used chemical ablation models to create olfactory nerve injury of varying severity and permanence. Franceschini et al. induced dorsomedial OE necrosis using dichlobenil in immune deficient mice, leading to irreversible damage of the underlying mucosa [46]. Lee et al. generated widespread olfactory neuronal loss in mice through systemic administration of 3-MI [47], while Park et al. produced transient anosmia in rats by applying Triton X-100 directly to the nasal cavity [48]. These models differ in the extent of epithelial destruction and the natural regenerative capacity of the olfactory epithelium, factors that influence the observed outcomes of stem cell therapy.

#### 4.3.2. Stem Cell Sources and Characteristics

Mesenchymal stem cells (MSCs), derived from bone marrow, adipose tissue, or umbilical cord, have been widely studied in experimental anosmia model. Adipose tissue-derived stem cells (ASCs) were isolated from rat adipose tissue and administrated to promote neurogenesis and endothelial repair [45]. Human umbilical cord blood CD133^+^ stem cells (HSCs) were delivered to immunodeficient mice and demonstrated the ability to migrate to injured areas and support olfactory epithelium regeneration [46]. Neural stem cells (NSCs) derived from the olfactory bulb of GFP-transgenic mice were used to directly replenish lost neuronal populations [47,49]. Bone marrow-derived MSCs (BMSCs) were transplanted locally in a rat model, where they secreted neurotrophic factors such as NGF and BDNF, facilitating neuroregeneration and epithelial repair [48]. These findings indicate that both multipotent MSCs and NSCs can contribute to olfactory epithelium regeneration.

Previous reports suggested that transplanted MSCs might differentiate into OSNs and endothelial cells. However, accumulating evidence indicates that such direct transdifferentiation of mesoderm-derived MSCs into ectodermal olfactory lineages (OSNs, GBCs, HBCs) remains highly controversial and likely limited in vivo [34,35]. Instead, the predominant mechanism by which MSCs contribute to olfactory epithelium regeneration is through paracrine signaling. MSCs secrete neurotrophic factors, cytokines, extracellular matrix proteins, and EVs that stimulate the proliferation and differentiation of endogenous olfactory progenitor cells. These paracrine effects explain observed improvements in epithelial thickness and food-finding behaviors, even in the absence of direct MSC integration into the olfactory epithelium. Therefore, MSCs should be considered as important modulators of the regenerative microenvironment rather than as direct neuronal precursors.

#### 4.3.3. Administration Routes of Stem Cells and Timing

The delivery route and timing of stem cell administration also varied. Systemic intravenous injection was employed for ASCs and HSCs, relying on their intrinsic migratory capacity to reach injured epithelium [45,46]. In contrast, local delivery via direct intranasal administration or injection near the olfactory epithelium was used for NSCs and BMSCs, achieving higher local cell concentrations and more efficient engraftment [47,48,49]. Repeated dosing, as in the ASCs study where injections were performed on consecutive days, appeared to enhance cell survival and integration compared to single-dose treatments [45].

#### 4.3.4. Functional and Histological Outcomes of Stem Cells

The reported outcomes demonstrated varying degrees of structural and functional recovery after stem cell therapy. Histological analyses consistently showed increased epithelial thickness and restoration of OSNs, indicated by olfactory marker protein (OMP)-positive staining in ASCs transplanted groups [45]. Molecular assessments revealed elevated levels of neurotrophic factors such as nerve growth factor (NGF) and brain derived neurotrophic factor (BDNF) following BMSCs or ASCs therapy, suggesting paracrine support for neuronal survival and differentiation [48]. Functional recovery was assessed using behavioral food-finding tests in mice and rats or electro-olfactogram recordings in the dichlobenil model, with HSC- or NSC-transplanted animals generally showing earlier and more robust responses compared to controls [46,47,49]. Engrafted ASCs differentiated into OSNs and endothelial cells [45], whereas human CD133^+^ HSCs contributed to chimeric cell population supporting tissue regeneration [46]. Green fluorescent protein (GFP)-positive NSCs were directly incorporated into the neuroepithelium and survived for several weeks post-transplantation [47,49].

## 5. MSCs-Derived Secretome and Extracellular Vesicles as Cell-Free Therapeutic Strategy

Although direct stem cell transplantation has demonstrated potential in promoting olfactory epithelium regeneration, it faces several biological and clinical limitations. Transplanted cells often exhibit poor survival and engraftment in injured tissue, face risks of immune rejection or tumorigenesis, and require complex manufacturing and delivery procedures [42,50]. Furthermore, long-term integration of transplanted neurons into existing olfactory circuits remains uncertain. These challenges have driven the exploration of cell-free alternatives that leverage the regenerative capacity of stem cells without introducing living cells into the host.

A growing body of evidence suggests that much of the therapeutic benefit of stem cell is mediated predominantly through paracrine mechanisms—via secreted bioactive molecules and EVs—rather than by direct neuronal replacement. The stem cell secretome comprises a complex mixture of neurotrophins (e.g., BDNF, NGF, vascular endothelial growth factor (VEGF)), anti-inflammatory cytokines (e.g., IL-10, TGF-β), extracellular matrix proteins, and regulatory microRNAs (e.g., miR-124, miR-133b, miR-21) [51]. These bioactive cargos modulate the host tissue microenvironment by enhancing survival of endogenous OSNs, stimulating basal stem cell proliferation, reducing inflammation, and promoting angiogenesis. EVs, particularly exosomes, serve as natural carriers of these molecules, allowing for efficient intracellular communication and delivery of regenerative signals [52]. For instance, miR-124 has been implicated in neuronal differentiation, VEGF supports vascular repair and survival of regenerating neurons, and IL-10/TGF-β restores epithelial homeostasis by reducing pro-inflammatory signaling [51,53]. Compared to MSCs transplantation, MSC-derived secretome and EV-based therapies offer several advantages including improved safety profile, standardization and storage, ease of delivery, low possibility of immune rejection, and no risk of aneuploidy or vascular occlusion [42,53].

**Figure 3:**
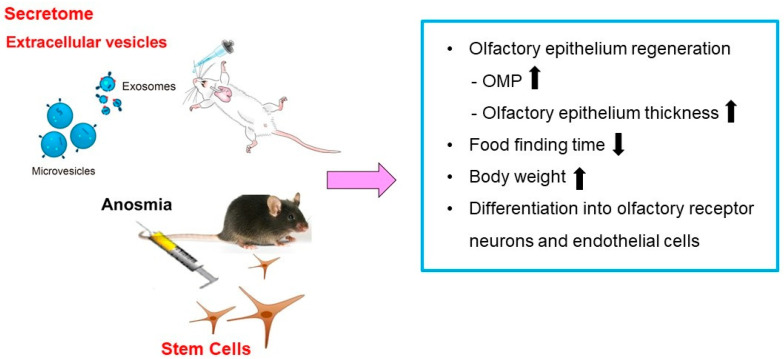
Effects of stem cell and stem cell-derived secretome or extracellular vesicles (EVs) on olfactory epithelium regeneration. Stem cells transplantation and administration of stem cell-derived secretome or EVs have been shown to promote olfactory epithelium regeneration, as evidenced by increased expression of olfactory marker protein (OMP) and enhanced epithelial thickness. These regenerative effects are associated with decreased food-finding time and increased body weight. Furthermore, stem cells may differentiate into olfactory receptor neurons and endothelial cells.

Recent studies have demonstrated that the administration of secretome or EVs derived from MSCs is as effective as the stem cells themselves in the suppression of allergic airway inflammation [53,54,55]. However, direct application of MSC-derived secretome or EVs for olfactory epithelium repair in anosmic animal models remains to be elucidated. Broadly related studies in other more accessible neural injury models support this cell-free approach (Table 3). Yavuz et al. reviewed applications of MSCs-derived EVs in nerve regeneration, demonstrating their capacity to promote neurite outgrowth, reduce inflammation, and enhance functional recovery across multiple animal models [56]. Although not specific to olfactory tissue, similar mechanisms including anti-inflammatory cytokines, neurotrophin-rich cargo, and miRNA-mediated signaling could be harnessed in anosmia models. Furthermore, studies on olfactory mucosa MSC-derived conditioned mediums have shown beneficial effects on peripheral nerve regeneration in rats, including improved motor and sensory recovery, indicating that secretome from olfactory-derived stem cells carries immunomodulatory and regenerative factors [57]. These findings suggest that secretome or EV-based therapies may offer comparable therapeutic efficacy to stem cells in promoting olfactory epithelium regeneration in anosmia models (Figure 3). Furthermore, intranasal administration of secretome or purified EVs derived from MSCs or NSC could be evaluated for the reduction of epithelial inflammation and apoptosis, enhancement of OMP positive neuron regeneration, acceleration of functional recovery, and identification of effective bioactive cargo such as miR-124, BDNF, NGF, and anti-inflammatory cytokines. Future investigations should focus on identifying key functional cargos within EVs and optimizing intranasal delivery strategies to maximize therapeutic outcomes. By shifting emphasis from cell replacement to paracrine and EV-mediating signaling, cell-free therapy may provide a safer and more standardized approach for clinical translation.

## 6. Conclusions

Endogenous regeneration, stem cell transplantation, and cell-free secretome/EV therapies represent complementary strategies, each with unique advantages and limitations (Table 4). Accumulating evidence supports secretome and EV-based therapy as a viable, safer, and more controllable alternative to stem cell transplantation for olfactory epithelium regeneration. Future studies integrating bioengineering approaches to enhance EV cargo specificity, improve delivery efficiency to the olfactory mucosa, and combine these therapies with endogenous stem cell activation may advance their translation into clinical practice.

## Figures and Tables

**Table 1:** Common chemical agents used in anosmia models.

Agent	Route	Target Cells	Mechanism of Injury	Regeneration Potential	Notable Features
Methimazole	IP	OSNs, basal cells	Apoptosis via oxidative stress	High	Consistent, reversible, OE-specific
Zinc sulfate	IN	Epithelial cells	Necrosis via oxidative damage	Variable	Inconsistent damage, affects other nasal tissues
Dichlobenil	IN	Sustentacular cells	Disruption of cell structure	Moderate	Targets non-neuronal cells, less widely used
3-Methylindole	IP	OSNs, basal cells, Clara cells	Reactive metabolites, inflammation	Moderate to high	Severe acute epithelial damage

IN, intranasal; IP, intraperitoneal; OSNs, olfactory sensory neurons.

**Table 2:** Summary of exogenous stem cell therapy studies for olfactory epithelium regeneration.

Study (Year)	Animal Model	Stem Cell	Delivery Route	Outcome Measures	Key Findings
Kim et al. (2009) [45]	Rats, Nerve transection	ASCs	IV	Histology (OMP, PCNA)	Partial structural recovery, differentiation into OE neurons and endothelial cells
Franceschini et al. (2009) [46]	Mice, Dichlobenil	HSCs	IV	Histology, FISH chimerism analysis, electro-olfactogram	Partial structural and functional recovery
Lee at al. (2010) [47]	Mice, 3-methylindole	NSCs	Transnasal	Food-finding test, histology (OMP)	Improved survival and faster olfactory recovery
Jo et al. (2015) [48]	Rats, Triton X-100	BMSCs	Transnasal	Food-finding test, histology (OMP)	Increased NGF and BDNF expression, improved OE thickness
Hazir et al. (2025) [49]	Mice, 3-methylindole	NSCs	Intranasal	Food-finding test, Histology (OE thickness, OMP+ neurons)	Behavioral improvement and epithelial regeneration

ASCs, adipose tissue-derived stem cells; BDNF, brain neurotrophic factor; BMSCs, bone marrow-derived mesenchymal stem cells; HSCs, human umbilical cord blood stem cells; IV, intravenous; NGF, nerve growth factor; NSCs, neural stem cells; OMP, olfactory marker protein; OE, olfactory epithelium; PCNA, proliferating cell nuclear antigen.

**Table 3:** Summary of secretome- and extracellular vesicles (EVs)-based therapy for olfactory epithelium regeneration.

Potential Study Model	Secretome/EVs	Delivery Route	Expected Outcome Measures	Rationale/Precedent
3-MI anosmia	Olfactory MSC-CM	Intranasal	Restore OMP+ neuron density, reduce epithelial thinning, improved FFT	Based on Alvites et al. (2022) [58] olfactory MSC CM in peripheral nerve repair
3-MI anosmia	NSC-derived exosomes	Intranasal	Modulate inflammation, promote basal cell proliferation, accelerate OSN restoration	Builds on Yavuz et al. (2024) [57] nerve EV regenerative mechanisms
Methimazole anosmia	miRNA-enriched EVs (miR-124, miR-133b)	Intranasal	Increase neurogenic miRNA expression, new synaptic markers, behavioral recovery	Based on Xin et al. (2013) [53] miRNA transfer in neural cells

CM, conditioned media; EVs, extracellular vesicles; FTT, food finding time; MI, methylindole; MSC, mesenchymal stem cell; NSCs, neural stem cells; OMP, olfactory marker protein; OSN, olfactory sensory neuron.

**Table 4:** Comparative overview of therapeutic strategies for olfactory epithelium regeneration.

Approach	Efficacy	Safety	Scalability	Translational Challenges
Endogenous regeneration (HBCs, GBCs)	Effective in mild to moderate injury; limited under aging or chronic inflammation	High (native cell source)	Intrinsic but declines with age/disease	Insufficient in severe or irreversible damage
Exogenous stem cell transplantation	Promotes OE thickening, OSN recovery, functional improvement in animal models	Risk of immune rejection, poor engraftment, tumorigenesis	Complex cell isolation, expansion, and quality control	Long-term neuronal integration uncertain; regulatory and ethical hurdles
Secretome/EV-based therapy	Comparable regenerative effects via paracrine cargo; enhances neurogenesis and reduces inflammation	High (cell-free, low immune risk)	Amenable to standardization, storage, and off-the-shelf use	Identification of key functional cargo; optimization of intranasal delivery

EVs, extracellular vesicles; GBCs, globose basal cells; HBCs, horizontal basal cells; OE, olfactory epithelium; OSN, olfactory sensory neuron.

## Data Availability

Not applicable.
